# A portable trap with electric lead catches up to 75% of an invasive fish species

**DOI:** 10.1038/srep28430

**Published:** 2016-06-24

**Authors:** Nicholas S. Johnson, Scott Miehls, Lisa M. O’Connor, Gale Bravener, Jessica Barber, Henry Thompson, John A. Tix, Tyler Bruning

**Affiliations:** 1 U. S. Geological Survey, Great Lakes Science Center, Hammond Bay Biological Station, 11188 Ray Road, Millersburg, MI 49759, USA; 2 Fisheries and Oceans Canada, Great Lakes Laboratory for Fisheries and Aquatic Sciences, 1219 Queen Street, East Sault Ste. Marie, CA, ON, P6A 2E5; 3 Fisheries and Oceans Canada, Sea Lamprey Control Centre, 1219 Queen Street, East Sault Ste. Marie, CA, ON P6A 2E5; 4 U. S. Fish and Wildlife Service, Marquette Biological Station, 3090 Wright St., Marquette, MI 49855, USA.

## Abstract

A novel system combining a trap and pulsed direct current electricity was able to catch up to 75% of tagged invasive sea lamprey *Petromyzon marinus* in free-flowing streams. Non-target mortality was rare and impacts to non-target migration were minimal; likely because pulsed direct current only needed to be activated at night (7 hours of each day). The system was completely portable and the annual cost of the trapping system was low ($4,800 U.S. dollars). Use of the technology is poised to substantially advance integrated control of sea lamprey, which threaten a fishery valued at 7 billion U.S. dollars annually, and help restore sea lamprey populations in Europe where they are native, but imperiled. The system may be broadly applicable to controlling invasive fishes and restoring valued fishes worldwide, thus having far reaching effects on ecosystems and societies.

Invasive species cost the global economy billions to trillions of U.S. dollars annually^1^,^2^, degrade ecological function and cultural opportunities^3^, and are posed to inflict greater damage during the next century given continued globalization and climate change^4^,^5^. Aquatic invasive fishes can be particularly damaging^6^ and difficult to control because the medium in which they live constrain methods to detect, monitor, remove, and modulate their behavior^7^,^8^,^9^,^10^. While methods exist to control some invasive fishes^11^,^12^,^13^, they can pose unknown or unacceptable risks to non-target species^14^, and are generally less effective and diverse than methods to control terrestrial pests.

Resource managers are often mandated to protect valued fishes while providing water for human use^15^,^16^ in addition to managing risks from invasive species. For example, hydropower is a renewable source of about 20% of the electricity used worldwide^17^,^18^ and impoundments provide water critical for human consumption and crops^19^. However, dams and impoundments disrupt connectivity of watersheds and natural flow regimes^20^,^21^, and therefore negatively influence ecosystem function and fish populations^22^,^23^. Fishes that reproduce in rivers and feed in the oceans (anadromous) are most at risk from fragmentation because of the need to surpass barriers while migrating to and from the ocean^24^.

Control of invasive fishes and restoration of valued fishes could be improved if a technology existed to guide their migration; in some cases invasive fishes could be guided into traps for removal and in other cases valued fishes could be guided to safe passage around dams. Many tactics already exist to guide fishes, but most are ineffective, costly, difficult to modify after construction, or cause an undesirable change in the waterway^25^,^26^. We conceptualized that because many fishes are electroreceptive^27^ and avoid electric fields^28^, pulsed direct current electricity could function as a non-physical means to guide fishes for control and restoration. Furthermore, if the device was easy to install and could be seasonally deployed, it could be used as a rapid response tool for emerging invasions or restoration needs in remote locations without altering streamflow.

We tested our conceptual model on adult sea lamprey *Petromyzon marinus* during their upstream spawning migration. The sea lamprey is highly invasive in the Laurentian Great Lakes^29^ where it threatens a fishery valued at 7 billion dollars annually^30^, but is listed as imperiled in much of Europe where it is ecologically and culturally important^31^,^32^. Furthermore, lamprey species worldwide are in decline and numerous efforts are underway to improve passage at dams^31^. Lampreys are among the earliest vertebrates species for which the capabilities of electroreception have been proposed^33^ and sea lamprey exhibit behavioral and neuroendocrine responses to low voltage electric fields^34^,^35^. Our study was in the Laurentian Great Lakes basin, so our goal was to develop a trap that could be installed seasonally in remote locations to remove adult sea lamprey before they spawn. A field of pulsed direct current induced by electrodes vertically oriented in the water column (electric lead) was used to guide sea lamprey to the trap, but the same approach could be used to guide valued lamprey species toward fish passage devices. Vertical electrodes offered several advantages over electrodes placed on the stream bottom including ease of installation, reduced cost, and a consistent electric field throughout the water column. While experiments conducted in semi-natural conditions showed that this type of electric lead can guide sea lamprey to traps^36^, an outstanding knowledge gap was whether results could be replicated in a completely natural setting with free-ranging sea lamprey. We tested the trap with electric lead in two sea lamprey infested streams, while tracking movement of sea lamprey and non-target fishes around the system using telemetry (passive integrated transponders; PIT). If successful, similar electric fields may be broadly applicable for control or restoration of fishes.

## Results

### Electric lead increases sea lamprey trap capture

**Figure 1:**
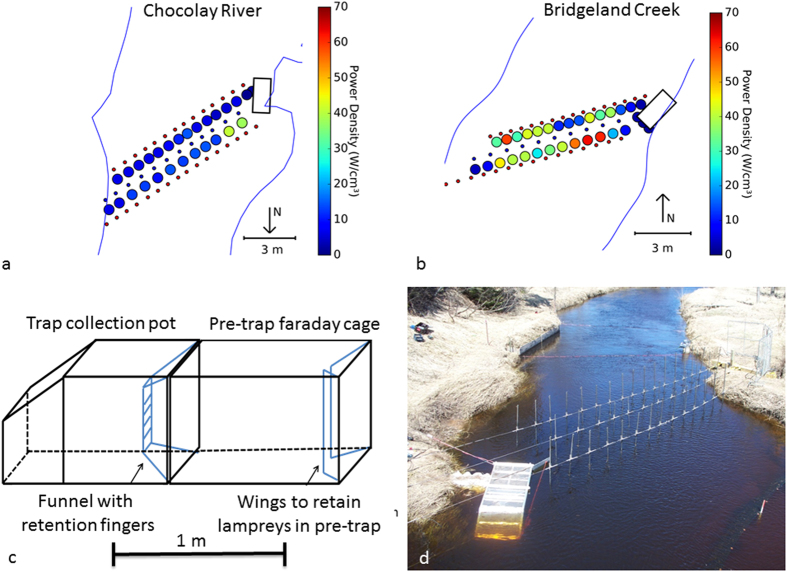
Trap and electric lead used to catch invasive sea lamprey. (**a**) Overhead perspective the portable trap and electric lead used in the Chocolay River, Michigan, to catch sea lamprey. Rectangles illustrate the location of the free-standing trap. Small red dots illustrate the location of positive electrodes and small blue dots illustrate the location of negative electrodes. Coloring in the large circles between the lines of electrodes illustrates the power density of the electric field at sampling locations. (**b**) Overhead perspective the portable trap and electric lead used in Bridgeland Creek, Ontario, to catch sea lamprey. (**c**) Illustration of the free-standing trap to which the electric lead guided sea lamprey. (**d**) Picture of the trap and electric field in the Chocolay River.

We deployed the trap and electric lead under completely natural conditions in the Chocolay River, MI, USA (Fig. 1), and operated the electric lead every other night to determine how many more sea lamprey were captured when the electric lead was on. Consistent with previous experiments^36^, the trap captured more sea lamprey (sea lamprey captured = 83, 48 males, 35 females) during nights when the electric lead was on than during nights when the electric lead was off (sea lamprey captured = 36, 18 males, 18 females). However, catching more sea lamprey when the electric lead was on did not provide unequivocal evidence that the electric lead guided the sea lamprey to the trap because more sea lamprey may have been migrating upstream during nights when the electric lead was on. We addressed this uncertainty by tracking the movement of PIT-tagged sea lamprey around the trap when the electric lead was on and off. During nights when the electric lead was on, 33% of PIT-tagged sea lamprey moving upstream were captured whereas only 2% of sea lamprey moving upstream were captured when the electric lead was off (logistic regression, z-value = 3.01, df = 94, p-value = 0.003; Supplementary Tables 1 and 2). When the electric lead was off, 96% of PIT-tagged sea lamprey escaped upstream of the trap, whereas only 22% escaped upstream of the trap when the electric lead was on (z-value = −5.49, df = 93, p-value < 0.001; Supplementary Table 2). When the electric lead was on, 46% of PIT-tagged sea lamprey returned downstream after encountering the system, meaning that those sea lamprey had another chance of being captured on a later date if they moved upstream.

### Portable trap with electric lead catches up to 75% of sea lamprey

A limitation of the experiment conducted in the Chocolay River was that the electric lead was not operated every night and sea lamprey escaped upstream when it was off. To determine the percentage of sea lamprey that could be removed if the electric lead was operated daily, the portable trap and electric lead was deployed in Bridgeland Creek, ON, Canada (Fig. 1), during two sea lamprey migratory seasons to catch free-ranging sea lamprey and PIT-tagged sea lamprey released downstream of the trap. During the first season, the portable trap with electric lead captured 2,440 free-ranging sea lamprey (1,452 males, 988 females). Of the 422 PIT-tagged sea lamprey (mean length 49 cm) that approached the electric trap over five release dates, 58% were captured in the trap, 42% escaped upstream of the trap, and less than 1% were neither captured nor escaped upstream (Supplementary Table 3). During the second season, the portable trap with electric lead captured 1,213 free-ranging sea lamprey (701 males, 512 females). Of the 565 PIT-tagged sea lamprey (mean length 48 cm) that approached the electric trap over six release dates, 75% were captured in the trap, 20% escaped upstream of the trap, and 5% were neither captured nor escaped upstream (Supplementary Table 3).

### Long sea lamprey were more likely to be captured in the trap with electric lead

**Figure 2:**
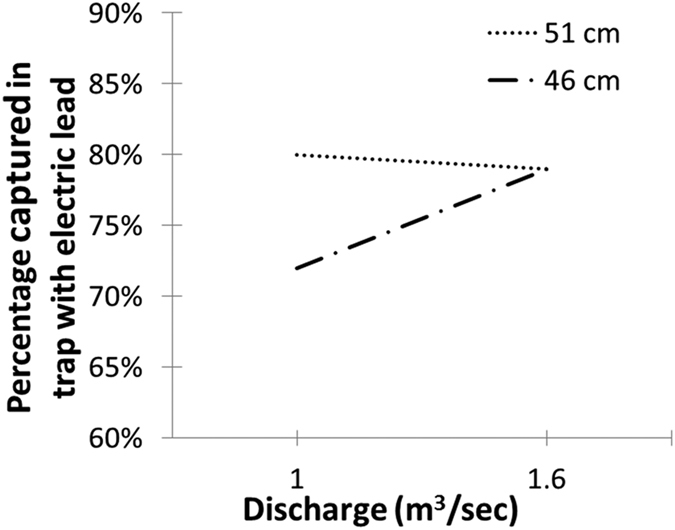
Model predicted percentage of PIT-tagged sea lamprey captured in a trap with electric lead in Bridgeland Creek, Ontario, during 2015 as a function of discharge and the length of the sea lamprey. During experimentation, discharge varied between 1.0 and 2.0 m^3^/sec; plotted are the 25^th^ and 75^th^ percentiles (1.0 and 1.6 m^3^/sec). Length of PIT-tagged sea lamprey varied between 31–59 cm; plotted are the 25^th^ and 75^th^ percentiles (46 and 51 cm). Percentage of PIT-tagged sea lamprey captured during 2014 was about 20% lower, but the same relationship between discharge and sea lamprey length was observed.

Not all sea lamprey that encountered the trap with electric lead in Bridgeland Creek were captured, so we investigated biological and environmental characteristics that might influence if a sea lamprey was captured in the trap or escaped upstream. Because we knew the sex and length of all PIT tagged sea lamprey released and knew which sea lamprey approached the trap with electric lead when it was on, we determined whether sex or length of the sea lamprey or whether stream discharge the night they approached the trap explained if the sea lamprey evaded capture. We also included year of release to evaluate the potential impact of adding a 25 cm fiberglass wing to the trap during the second season (see methods). PIT-tagged sea lamprey were about 20% more likely to be captured in the trap with an electric lead during the second season (logistic regression model here and below; estimate = 0.83, t_955_ = 5.11, p < 0.001), potentially resulting from the addition of a 25 cm fiberglass wing to the trap during year two (see methods). During both years, sea lamprey that were longer than the median length (48 cm) were more likely to be captured (estimate = 0.22, t_955_ = 2.20, p = 0.028), and sea lamprey were more likely to be captured when discharge was higher than the median observed discharge (estimate = 6.84, t_413_ = 1.92, p = 0.054). However, sea lamprey length and discharge interacted (estimate = −0.13, t_413_ = −1.34, p = 0.063) such that at high discharge, long and short sea lamprey had nearly the same probability of being captured (Fig. 2). During the second season, when the electric trap lead was only activated at night, 90% of sea lamprey moved at night. About 20% of sea lamprey that escaped upstream of the trap during the second season did so when the electric lead was deactivated during the day.

Water temperature and ambient conductivity were not included in the analysis because they were negatively correlated with discharge (correlation coefficient = −0.60, −0.65, respectively). Julian date was not included as a possible predictor because all sea lamprey released were sexually immature. Sex was evaluated, but removal of the sex effect reduced AIC by 2, whereas removal of any other effect resulted in an AIC higher than the full model. Therefore, results from the model without sex were reported. Interactions of length and discharge with sex were not considered because sex was not significant as a main effect in the model. All models evaluated and the final model reported here showed no evidence of overdispersion or nonlinearities.

### Non-target species were minimally impacted by the trap with electric lead

An effective invasive species trapping device is most likely to be useful if it minimally impacts non-target species. Therefore, at Bridgeland Creek we also evaluated the capture of non-target species in the trap with electric lead and a standard sea lamprey trap that was operated 40 m upstream to determine what non-target species were moving through the site. During the first season at Bridgeland Creek, the trap with electric lead captured 511 non-target fishes representing 12 different species, while a standard sea lamprey trap captured 1,354 non-target fishes representing 11 different species (Supplementary Table 4). During the second season, the trap with electric lead captured 1,262 non-target fishes representing 13 different species and the standard trap captured 557 non-target fishes representing12 different species (Supplementary Table 4).

Since 1999, ten non-target species have been consistently captured in the standard sea lamprey trap operated upstream of the trap with electric lead (Supplementary Table 5), so we evaluated if the catch of those non-target species in the standard sea lamprey trap was lower during years when the trap with electric lead was installed. During the first season, four of these species were captured in lower numbers than expected from previous years. During the second season, five of the ten species were captured in lower numbers than expected from previous years. When non-target catch from the trap with electric lead and the trap upstream were combined, all but one species (rainbow trout, *Oncorhynchus mykiss*) were captured at expected levels based on previous years, although only about five rainbow trout were captured on average per year. Most non-target fishes were removed from the trap and released alive, except during the first season, when overcrowding of sea lamprey and non-target fishes in the trap with electric lead caused significant mortality during nine nights (Supplementary Table 4; Supplementary Figure 1).

Because adult sea lamprey are primarily nocturnal, the electric lead was operated from 2200 to 0500 during most nights in Bridgeland Creek to reduce impacts to non-target species (see methods). During the second trapping season, we tracked movement of PIT-tagged rock bass (*Ambloplites rupestris*) to determine if non-target fishes captured in the standard sea lamprey trap upstream passed the electric lead while off or on (during the day or night). Rock bass were the most abundant non-target species observed in our experiment. Other non-target fishes were tagged, but not in great enough numbers to reveal their migratory patterns (Supplementary Table 6). Of 69 rock bass tagged and released below the trap with electric lead, 62% were detected below the trap with electric lead and 81% of those were detected above the trap with electric lead. Of the rock bass detected upstream of the electric lead, 95% moved past the system during the day when it was deactivated (Supplementary Table 6) supporting the assumption that diurnal species will move through the electrode array during the day when it is off.

Although the voltage gradient and power density between positive and negative electrodes was less than the threshold expected to cause injury^36^ (Fig. 1a,b), the voltage gradient close to the negative electrodes was higher because voltage gradients increase exponentially as distance to an electrode decreases. As such, we surveyed daily for dead and injured fishes around and downstream of the electric lead at Bridgeland Creek. During the first season, 26 dead fish were observed in or near the electric lead during visual assessments, including 17 dead sea lamprey and nine other unidentifiable fishes. During 2015, 11 dead fishes were observed in or near the electric lead; eight sea lamprey, two rainbow trout, and one common shiner *Luxilus cornutus*. Whether the dead sea lamprey and rainbow trout observed downstream of the electric lead died of natural causes after spawning or from exposure to the electric field is unknown.

### Cost of portable trap with electric lead was low

A fish guidance device is most likely to be useful if it is cost effective relative to other technologies, so total cost (U.S. dollars) to operate the trap with electric lead at Bridgeland Creek was calculated. The average annual cost to staff a sea lamprey trap currently ranges between $5,000 and $15,000 (U.S. Dollars). Here, we specifically calculated the annual cost of the trapping device; comparing the cost of the trap with electric lead to traps traditionally used to catch sea lamprey. The trap with electric lead cost $45,000 and dividing the cost by the estimated life expectancy (15 years), yielded an annual expense of $3,000. Multiplying the hours required to seasonally deploy, clean, and seasonally decommission the trap with electric lead (90 hours) by a technician hourly pay rate ($20) yielded an average yearly staff cost of $1,800. Taken together, the total annual cost for the trap with electric lead was $4,800, not including staff costs for operating the trap itself.

## Discussion

The current study builds on previous work which showed that the electric guidance system was effective at increasing sea lamprey encounter rates with traps in laboratory and small-scale stream experiments over short time scales (single nights)^36^. However, initial work did not determine if (1) if the device could be quickly deployed in completely natural streams where sea lamprey control was required, (2) if performance could be maintained during high flows, and (3) how operation over multiple weeks impacted non-target species. The current study addresses these remaining critical knowledge gaps and directly transferred our research to the sea lamprey control program by conducting the experiments with sea lamprey control agents in sea lamprey infested streams.

Here, by pairing a non-physical electric lead with a portable trap, we were able to remove up to 75% of the tagged invasive sea lamprey in a natural stream using a single device that was seasonally deployed and cost effective relative to most traditional physical structures used to catch sea lamprey. Physical devices such as screens or concrete devices have been used to direct fishes to passage devices or traps, but they physically alter the waterway, can accumulate debris, are difficult to modify after construction, and can be prohibitively expensive^37^,^38^. Non-physical cues such as light, sound, bubbles, CO_2_, chemosensory cues, can alter fish behavior without obstructing a waterway, and can be deployed where physical devices would cause an unacceptable change in a waterway^39^. However, many non-physical devices do not meet management targets and do not incorporate a trap to physically remove invasive species. When a trap is not incorporated, invasive species either spawn below the device, disperse to other habitats, or escape upstream of the device^39^. Currently, adult sea lamprey trapping relies on physical barriers that block sea lamprey migration, can be much more costly (ranging $50,000 to $1,000,000), and removal rates vary greatly among streams (ranging from 10% to 70%). For example, the mean removal rate achieved with the electric lead in 2014 and 2015 (67%) is very similar to that of the permanent trap on Bridgeland Creek, which is one of the most effective traps in the Great Lakes Basin (mean 70%; 1999–2013).

The electric field used here overcame many of the challenges of previous electric systems used to modify fish behavior. The first electric barriers to block sea lamprey in the Great Lakes used 110 V alternating current actuated through vertical electrodes suspended above the stream^40^. These devices were generally effective and easy to install and 162 barriers were built within 10 years for sea lamprey control^41^. However, these systems blocked all fishes and non-target mortality was high because alternating current was used^41^. During the 1980s, direct current electrical barriers were designed with horizontally mounted electrodes laid on the stream bottom^42^,^43^. However, these systems were decommissioned in subsequent years because they were not 100% effective, blocked non-target fishes, and the cost and logistics of seasonal deployment was daunting^42^. During this study, pulsed direct current was used instead of alternating current to reduce the probability of fish injury. Fishes were guided to a live trap, to remove invaders and release valued fishes. Furthermore, vertical electrodes were quickly installed without major stream modification. From a physics standpoint, vertical electrodes produce an electric field that is often more effective for guiding fishes than horizontal electrodes because vertical electrodes produce a consistent voltage gradient throughout the water column; voltage gradient decreases near the surface of the water with horizontal electrodes placed on bottom. The electrode design used here was resilient to several flood events (Supplementary Figure 5). However, the vertical electrodes deployed from overhead lines in this study would have blocked boat traffic, could have been fouled by debris, and may only be practical to deploy in streams less than 2 m deep and 25 m wide. Our test streams were shallow and did not have boat traffic. The system was effective at removing invasive sea lamprey, could be deployed quickly and seasonally, and generally inflicted no injuries to fishes in small rivers. Deployment of bottom-mounted vertical electrodes would be required in deeper streams with boat traffic, with the only depth limitation being the depth at which the electrodes would be too long to practically deploy.

The trap with electric lead captured most sea lamprey in the stream, while also allowing passage of non-target species, unlike many other physical and non-physical barriers. As sea lamprey are primarily nocturnal^44^ and our objective was not to block or remove 100% of adult sea lamprey, we operated the electric lead primarily at night. The primary non-target species (rock bass) passed through while the electric lead was not operated during the day, while sea lamprey generally did not. However, some non-target species captured upstream of the trap are nocturnal, so small species such as brown bullhead *Ameiurus nebulosus* likely passed through the electric field during the night when it was activated. The voltage transferred to a fish depends in part on the voltage gradient in the water and fish length^28^ which explains our observation that smaller sea lamprey were more likely to escape upstream of the trap with electric lead. As such, longer nocturnally migrating fishes such as walleye (*Sander vitreus*; in the Great Lakes region) may be guided by the electric field similar to sea lamprey, but this has not been explicitly tested. Recently, a similar electric guidance system induced avoidance responses in several downstream migrating European fishes in laboratory environments^45^, so it is conceivable that many of the non-target fishes captured may have been guided to the trap by the electric field. Unfortunately, during nights when sea lamprey capture was high, mortality of non-target fishes captured in the trap was also high. We attribute this high mortality to overcrowding because it corresponded with nights of high sea lamprey catch and previous studies found that fishes exposed to the electric field had minimal injuries^36^. Mortality caused by overcrowding could be lessened by making the fish holding compartment larger. Taken together, the sensitivity of fishes to electric fields vary and the amount of electricity transferred to fishes varies according to their length^28^, so electric guidance devices may be tailored to select for different species; for example, invasive fishes such as Asian carps *Hypophthalmichthys sp.*, northern pike *Esox lucius*, rainbow trout, and valued fishes such as Pacific lamprey *Entosphenus tridentatus*, Pacific salmon *Oncorhynchus sp.*, and walleye.

Throughout the world, lampreys are valued for the ecological services they provide as well as their value as food^31^, but populations are in decline in part due to barriers that block their spawning migrations^32^. The electric guidance system tested here could be useful for effectively directing lamprey to bypass routes around dams. If lamprey bypass routes are not present, the guidance system and portable trap could be used to capture lamprey for transport and release upstream of dams or for use in propagation programs^32^.

In the case of sea lamprey control in the Laurentian Great Lakes, removing adult sea lamprey using traps with electric leads may be sufficient to achieve ecosystem restoration goals. To reduce lake-wide adult sea lamprey abundance to levels sufficient to achieve ecosystem restoration goals, removal of 59% of the Great Lakes spawning stock each year is suggested^46^,^47^. This can be achieved by increasing removal to 60% or better at existing trap sites and expanding trapping to additional streams^46^. The pulsed direct current trap lead used here has the potential to substantially increase sea lamprey removal rates at existing barrier-integrated traps at relatively low cost and enable trapping on most sea lamprey producing tributaries that are not currently trapped.

Integration of electric leads with other tactics could improve efficiency and reproductive suppression. The trap with electric lead could remove more sea lamprey if the trap was baited with synthesized sex pheromone^48^,^49^ (pull) and if chemosensory alarm cue was applied in conjunction with the electric lead^50^ (push). If 90% of adult sea lamprey were removed in a trap integrating pheromones, repellents, and pulsed direct current, and the males from the trap were sterilized and released back into the stream^51^, the ratio of sterile to normal males in the stream would equal 9:1 regardless of population size (assuming a 50:50 sex ratio), thereby yielding a total reduction in reproductive potential of 99%. This integrated approach could be highly effective for sea lamprey in free flowing rivers and be used as a model for invaders in both aquatic and terrestrial habitats.

## Methods

### Stream characteristics

The Chocolay River is a tributary to Lake Superior with discharge between 3 and 5 m^3^/sec during the spring (Supplementary Figure 2). An aluminum mesh trap (diamond-shaped with hole openings of 14 mm × 43 mm; Fig. 1c) was deployed 20 m downstream of Mangum Road along the right bank (looking upstream) in a straight stretch of stream glide habitat approximately 10–15 km from the river mouth (Supplementary Figure 2). At that site, the substrate was a mixture of gravel and sand and during baseflow conditions (discharge = 1.3 m^3^/sec), the width was 11 m and the depth and water velocity across the channel averaged 0.86 m (SD = 0.22 m) and 0.24 m/sec (SD = 0.09 m/sec), respectively (Supplementary Figure 3).

Bridgeland Creek is a tributary of the Thessalon River, Ontario, which drains into the North Channel of Lake Huron (Supplementary Figure 2) with spring discharge of between 3 and 5 m^3^/sec. The trap was deployed 40 m downstream of a standard sea lamprey barrier and trap constructed of screening material (Supplementary Figure 2). The trap with electric lead was of the same design as that illustrated in Fig. 1, except that during the second season a 25 cm fiberglass wing (constructed of T-bar fiberglass pedestrian walkway grating; bar width 3.8 cm with 1.3 cm gap between bars) was attached to the downstream corner of the trap on the side adjacent to the electric field at a 45° angle to reduce the number of sea lamprey escaping upstream of the electric field by swimming adjacent to the trap. At that site, the substrate was a mixture of cobble and rubble, and during baseflow conditions (discharge = 1.00 m^3^/sec), the width was 12 m and the depth and water velocity across the channel averaged 0.39 m (SD = 0.13 m) and 0.59 m/sec (SD = 0.35 m/sec), respectively (Supplementary Figure 4). Water temperature and conductivity in both streams were logged every hour during experimentation using a sonde (6920 V2 Multi-Parameter, YSI Inc, Yellow Springs, Ohio). Depth and water velocity in the electric field was measured using a flow meter and wading rod (Marsh-McBirney Flow Mate, Hach).

### Electric lead

At both sites, the electric lead consisted of three lines of electrodes supported by overhead cables. Electrode locations were georeferenced (Trimble GeoExplorer) and plotted on a stream map (Fig. 1). Electrodes hung from the middle overhead cable had negative polarization and electrodes hung from the outer two overhead cables had positive polarization. Overhead lines were parallel and separated by 1.25 m (as measured parallel to streamflow). This produced a symmetric electric field to trigger an avoidance response in both upstream and downstream moving fishes. The negative electrode line started at the left corner of the trap entrance and extended downstream at a 55° angle to the left bank. Positive electrodes on the downstream line were spaced 0.70 m apart, negative electrodes (n = 10) were spaced 0.90 m apart, and positive electrodes on the upstream line were spaced 0.58 m apart. Electrodes were 2.4 m sections of long stainless steel pipe with lead inserted into the bottom 0.50 m of each electrode to keep them from swinging off bottom during normal flow conditions. Positive electrodes had an outside and inside diameter of 3.8 cm and 3.4 cm, respectively. Negative electrodes had an outside and inside diameter of 2.6 cm and 2.2 cm, respectively. Positive electrodes weighed 7.8 kg and negative electrodes weighed 6.6 kg. Electrodes were energized with dual frequency pulsed direct current produced by a portable pulsator unit (Procom Systems, Wroclaw, Poland) and distributed in North America by Fishways Global LLC, Livonia, MI). The pulsator was programmed to produce a group (packet) of DC pulses consisting of five 1.8 ms pulses with four 8.2 ms off-periods in between for a total duration of 41.8 ms (duty cycle = 9%). The duration from the start of one group to the next was 100 ms.

### Operation of trap with electric lead

At the Chocolay River, the trap with electric lead was operated from 2200 to 0500 hours every other night starting April 29^th^ and continuing until June 24^th^, 2015. The electric lead was only operated during the night to minimize the impact to non-target fish migration, while still targeting most migratory sea lamprey. The electric lead was not operated and the trap was not checked during a flood event (May 26^th^ through June 3^rd^) because the trap could not be accessed (Supplementary Figure 5). At Bridgeland Creek, during 2014, the electric lead was operated from 2200 to 0500 hours each night from May 11^th^ to May 23^rd^. From May 23^rd^ until June 18^th^, the electric lead was operated continuously except when checking the trap and when power failed during June 2^nd^ from 0300 to 1000, 1700 on June 2^nd^ to 1000 on June 3^rd^, and 1700 on June 3^rd^ to 1000 on June 4^th^. May 23^rd^ was the first day water temperatures reached 16 °C, which is the temperature when sea lamprey are known to become active during the day^44^. During 2015, the electric lead was operated from 2200 to 0500 hours each night from April 27^th^ to June 26, with the exception of June 6^th^, 7^th^, 12^th^, and 13^th^, when power surges occurred disabling the electric lead during part or all of those nights (unable to be determined). During 2015, the electric lead was operated only at night during the entire operational period regardless of water temperature to determine if high sea lamprey exploitation rates observed during 2014 could be achieved with presumably less impact to non-target species. All traps used in the study were checked daily (except during the Chocolay River flood). Captured animals were sorted according to species. Captured sea lamprey were sexed and inspected for passive integrated transponders (PIT tags). Non-target catch was also inspected for PIT tags during 2015, counted, and immediately released. Experimental protocols involving the handling of fishes were carried out in accordance with United States federal guidelines for care and use of animals and were approved by the American Fisheries Society through the “Use of Fishes in Research Committee, 2014”^52^.

### Movements of PIT sea lamprey around the trap with electric lead

At least 12 hours prior to release, sexually immature sea lamprey were weighed and measured and then uniquely-encoded PIT tags (32 mm) were inserted in the abdomen through a 3 mm incision. Tagged sea lamprey were placed in release cages (1 m^3^) located 0.5 km and 1.5 km downstream of the trap on Chocolay River and Bridgeland Creek, respectively. Cage entrances were opened at 1600 hours for each release event. In Bridgeland Creek, the number of PIT-tagged sea lamprey (1) approaching within 20 m of the trap with electric lead, (2) moving downstream from the trap, and (3) escaping upstream of the trap was determined by placing two PIT tag detection antennas 20 m below and above the trap. A similar approach was used at Chocolay River except that one of the upstream PIT-tag detection antennas was not deployed and instead was placed at the trap entrance to quantify encounters with the trap entrance. Sea lamprey to be PIT-tagged were sourced from multiple streams because at times sea lamprey availability was limited. At the Chocolay River, PIT-tagged sea lamprey were initially captured in the Chocolay River and the Rock River, MI. At Bridgeland Creek, nearly all the PIT-tagged sea lamprey were sourced from the Cheboygan River, MI, except during the first year when sea lamprey were also sourced from the Bridgeland Creek and the Echo River during the first release date. See Supplementary Tables 1 and 3 for more details about source and release schedules for tagged sea lamprey.

Of the PIT-tagged sea lamprey that approached the trap with electric lead in Bridgeland Creek during both years, variability in the proportion of sea lamprey that were captured during both years was determined using generalized linear models assuming a binomial error distribution (logistic regression) where the probability of capture was explained by biological, chemical, and physical variables: (1) sex, because male and female sea lamprey may respond differently to electric fields, (2) length, because voltage change across a long sea lamprey would be greater than voltage changed across a short sea lamprey, (3) stream discharge, because at high stream discharges sea lamprey would be exposed to the electric lead longer because progress upstream would be slower and (4) the interaction between length and stream discharge, because short sea lamprey may be more likely to escape through the field, but they also have poorer swimming ability. The Akaike Information Criterion (AIC) based stepwise procedure described in Faraway (2005)^53^ was used to identify the most parsimonious set of predictors by eliminating predictors explaining little of the variability in the response.

### Impacts to non-target species

In Bridgeland Creek, capture of non-target species in the standard trap fished upstream of the trap with the electric lead during 2014 and 2015 were compared to historic captures of non-target species (1999–2013). For each species that was captured in the upstream trap in at least 11 of the past 13 years (1999–2013), the 95% confidence interval (α = 0.05) was calculated for the number of individuals from each species expected to be captured in the upstream trap in a given year. We determined if the number of individuals captured in the upstream trap by species during 2014 and 2015 was within the 95% confidence interval of the historic number captured. Captures of non-target species in the trap with electric lead were also recorded and a survey for injured or dead animals was conducted daily by walking from the trap with electric lead downstream 200 m.

During 2015, nets were deployed downstream of the trap with the electric lead to capture non-target species moving upstream. Any non-target species greater than 120 mm received a 23 mm PIT tag (Supplemental Table 6) through a 3 mm incision in the abdomen after being anesthetized with 0.004 mg/L clove oil. Tagged fishes were released 60 m downstream of the trap with electric lead after a recovery period of about 1 hour.

## Additional Information

**How to cite this article**: Johnson, N. S. *et al*. A portable trap with electric lead catches up to 75% of an invasive fish species. *Sci. Rep.* **6**, 28430; doi: 10.1038/srep28430 (2016).

## Supplementary Material

Supplementary Information
