# Supplementary material for: A portable trap with electric lead catches up to 75% of an invasive fish species
Source: Sci Rep. 2016 Jun 24;6:28430. doi: 10.1038/srep28430 (PMC4920034; doi:10.1038/srep28430)
Supplement: Supplementary Information [file srep28430-s1.pdf]

Supplemental Information

A portable trap with electric lead catches up to 75% of an invasive fish species

Nicholas S. Johnson[1*], Scott Miehls[1], Lisa M. O'Connor[2], Gale Bravener[3], Jessica Barber[4], Henry, Thompson[1], John Tix[1], Tyler Bruning[1]

[1]U. S. Geological Survey, Great Lakes Science Center, Hammond Bay Biological Station, 11188 Ray Road, Millersburg, MI 49759

[2]Fisheries and Oceans Canada, Great Lakes Laboratory for Fisheries and Aquatic Sciences, 1219 Queen Street, East Sault Ste. Marie, ON.

[3]Fisheries and Oceans Canada, Sea Lamprey Control Centre, 1219 Queen Street, East Sault Ste. Marie, ON P6A 2E5

[4]U. S. Fish and Wildlife Service, Marquette Biological Station, 3090 Wright St., Marquette, MI 49855

*Corresponding author: njohnson@usgs.gov

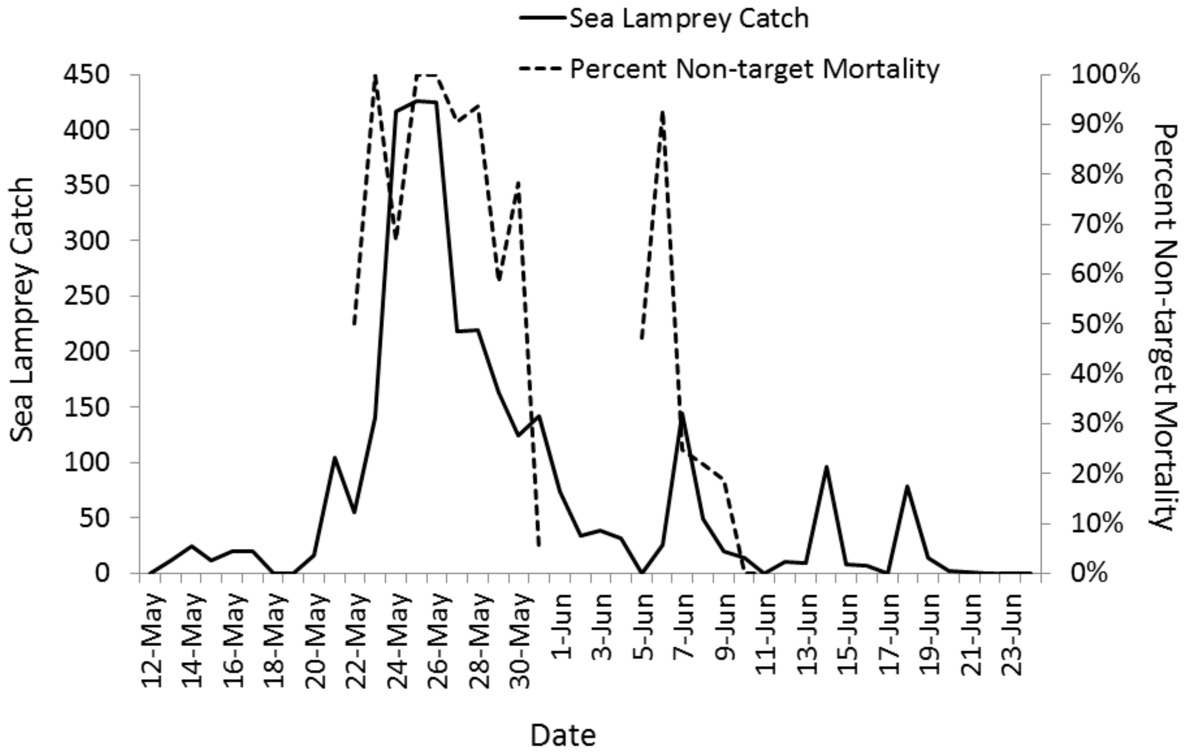

Supplementary Figure 1. In Bridgeland Creek, Ontario, during 2014, the number of sea lamprey captured in the trap with electric lead (solid line) and the percent of the total non-target catch that died prior to removal from the trap (dotted line). When zero non-targets were captured the percent non-target mortality is not included on the graph. During 2015, less non-target mortality was observed (**Supplementary Table 4**) presumably because fewer sea lamprey were captured (less crowding in the trap).

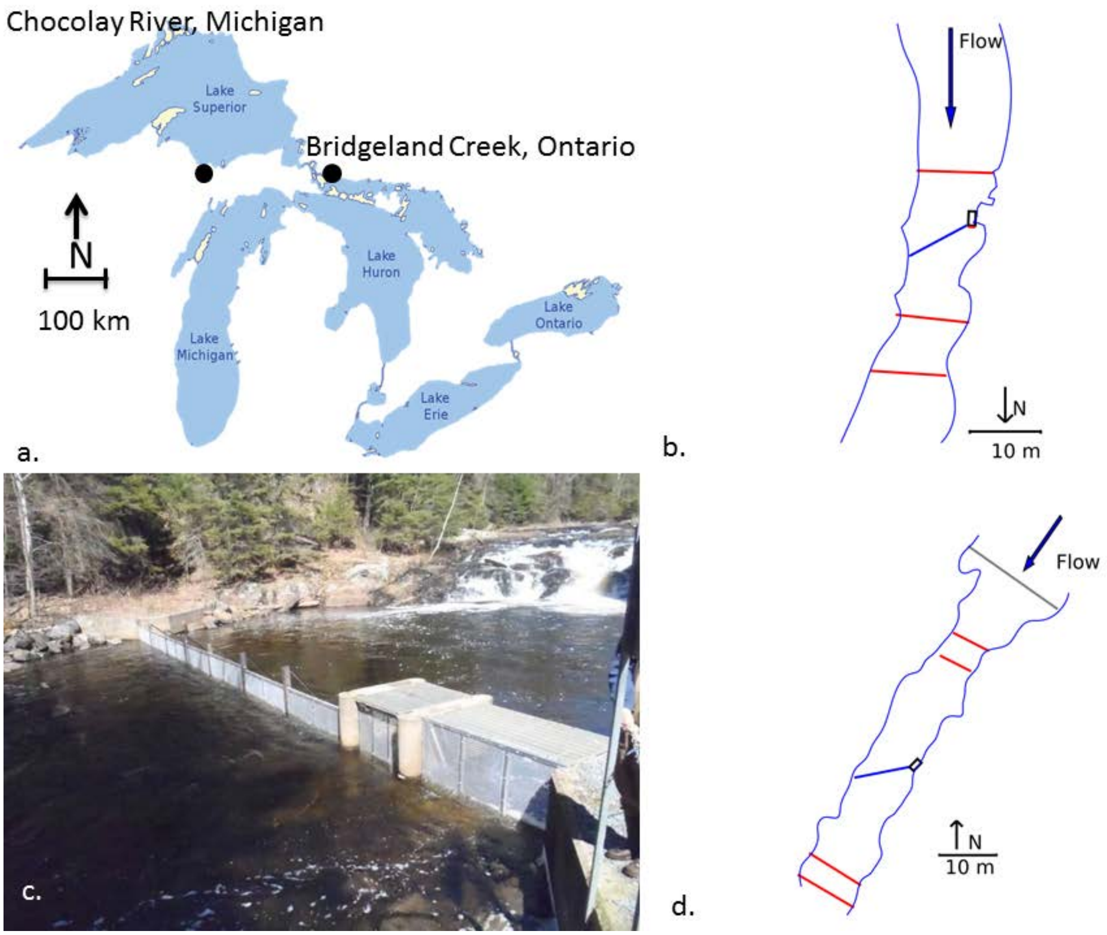

Supplementary Figure 2. **a.** Chocolay River is located in Michigan, USA, and is a tributary of Lake Superior. Bridgeland Creek is located in Ontario, Canada, and is a tributary to Lake Huron. **b.** Overhead perspective of Chocolay River highlighting the location of the trap (black rectangle and electric lead is angled blue line) and passive integrated transponder detection devices (PIT line; red color). Water flows from top to bottom **c.** Picture of the trap upstream of the trap with electric lead on Bridgeland Creek. **d.** Overhead perspective of Bridgeland Creek highlighting the location of the screen trap (thin black downstream of flow arrow), vertical electrode pulsed-direct current trap (black rectangle and electric lead is angled blue line), and passive integrated transponder detection devices (PIT line; red color). Water flows from top to bottom. Location map Great Lakes (https://en.wikipedia.org/wiki/Template:Location_map_Great_Lakes) is licensed under the Attribution-ShareAlike 3.0 Unported license. The license terms can be found on the following link: http://creativecommons.org/licenses/by-sa/3.0/".

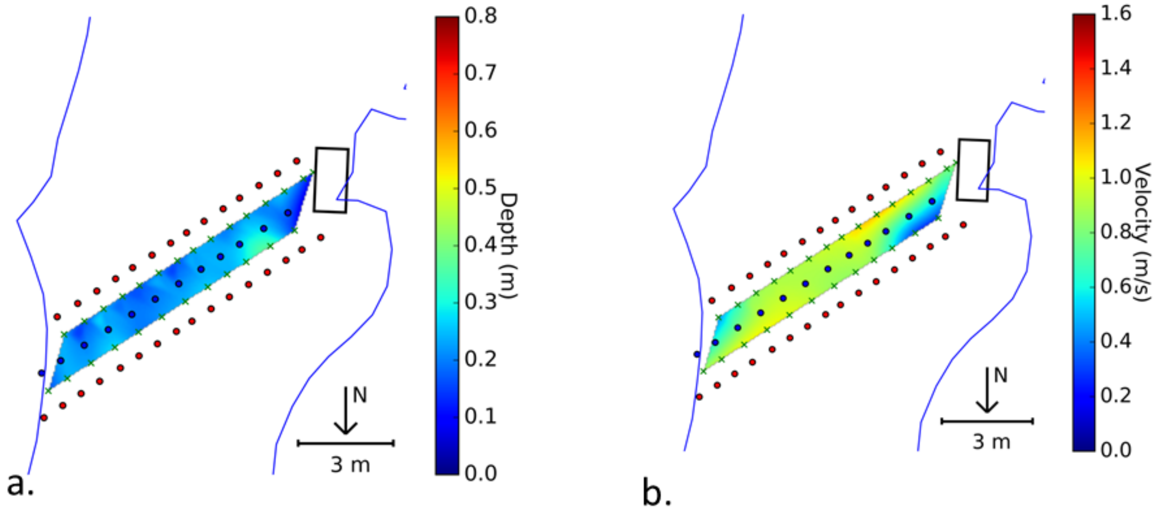

Supplementary Figure 3. Water depth (a) and velocity (b) within the electric lead field during baseflow conditions (~1.0 cms) at Chocolay River, Michigan. Red dots illustrate the position of positive electrodes and blue dots illustrate the position of negative electrodes. Color shading in the stream channel illustrates interpolated depth and velocity based on measurements between the electrode lines (indicated by small blue x's).

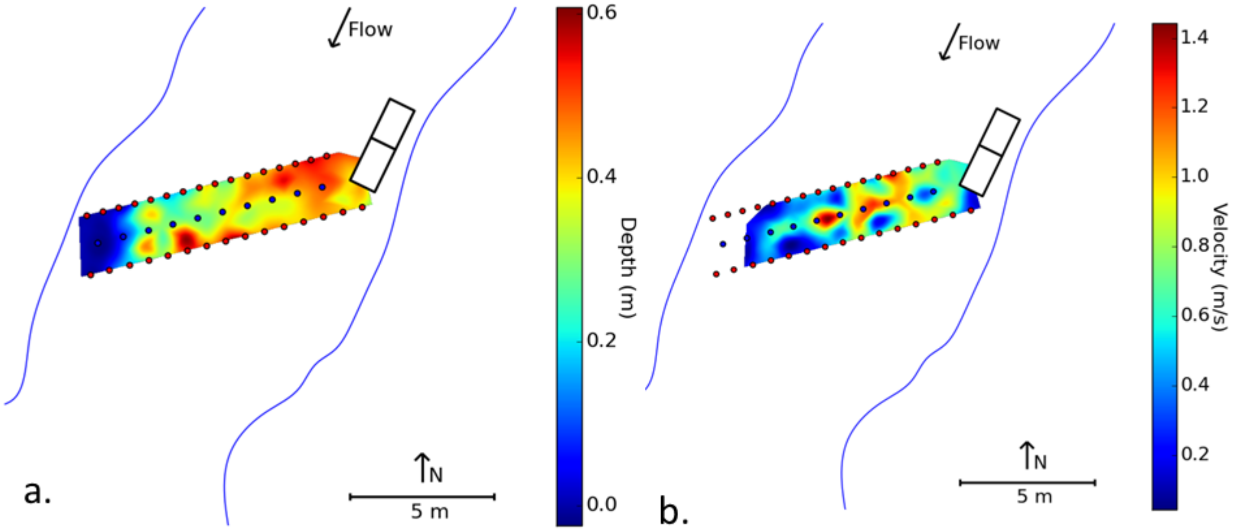

Supplementary Figure 4. Water depth (a) and velocity (b) within the electric lead field during baseflow conditions (~1.0 cms) at Bridgeland Creek, Ontario. Red dots illustrate the position of positive electrodes and blue dots illustrate the position of negative electrodes. Color shading in the stream channel illustrates interpolated depth and velocity based on measurements between the electrode lines.

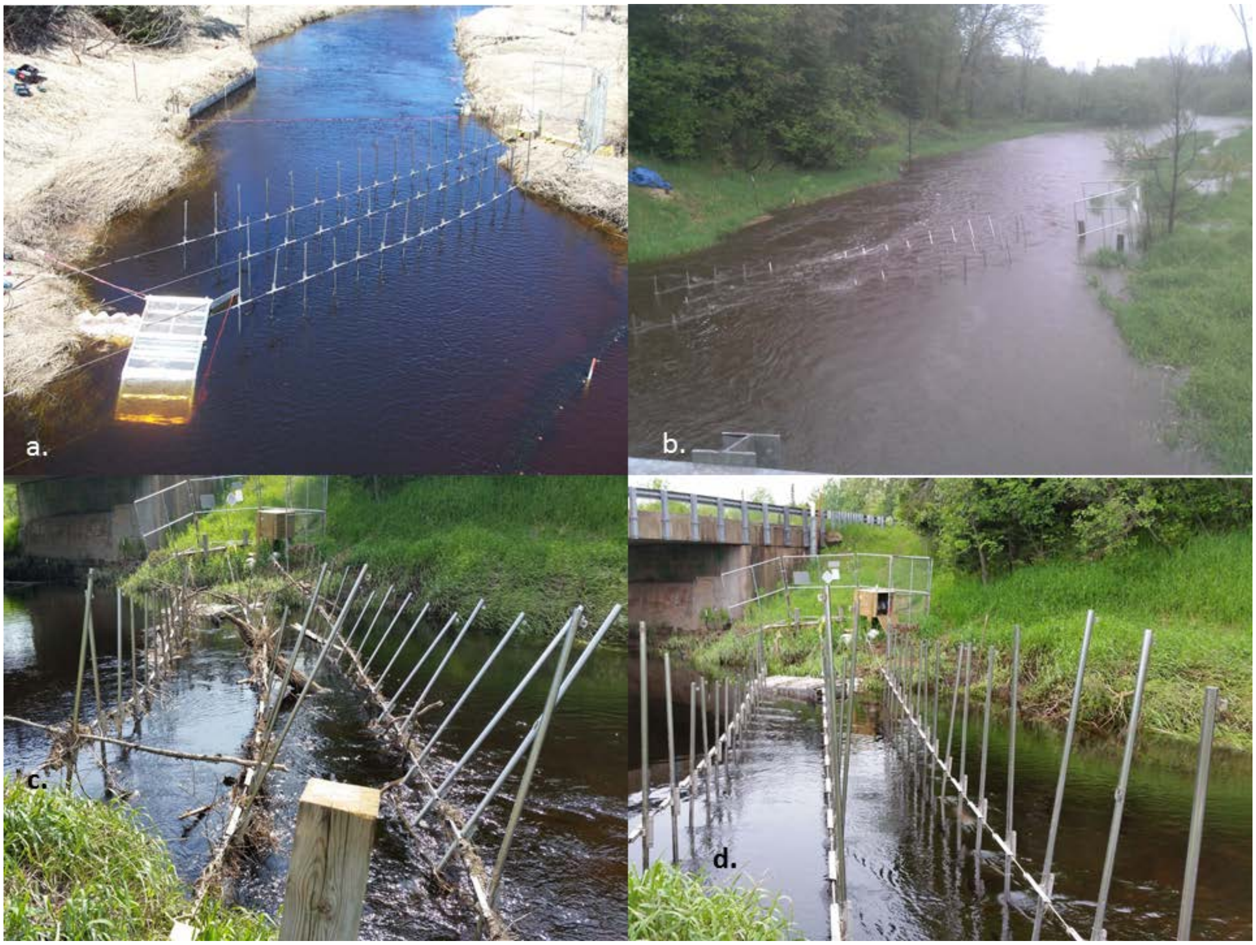

Supplementary Figure 5. a. Trap with electric lead and lead as deployed in Chocolay River, MI, during 2015 during bankfull discharge. **b.** Electric lead during a 5 year flood event during 2015. **c.** Electric lead after 5 year flood event. **d.** Electric lead after 4 technician hours of cleaning occurred after the flood event.

Supplementary Tables

Supplementary Table 1. The source and number of PIT-tagged sea lamprey released in the Chocolay River, MI during 2015, where 'nets' mean that the sea lamprey were previously captured in the fyke nets fished above the trap with electric lead in the Chocolay River, 'Electric' mean that the sea lamprey were previously captured in the trap with electric lead, and 'Rock' means that the sea lamprey were previously captured in a sea lamprey trap set in the Rock River, MI (a Lake Superior tributary about 50 km from the Chocolay River). Water temperature, water conductivity, and stream discharge measured at 2200 hours on the release data are also reported.

Release Date	Source	Number	VE-PDC	Temp °C	Conductivity (us/ms)	Discharge (m^3/sec)
7-May-15	Nets	23	On	17.1	180	1.8
7-May-15	Electric	15	On	17.1	180	1.8
7-May-15	Rock	2	On	17.1	180	1.8
8-May-15	Nets	23	Off	15.0	177	1.7
8-May-15	Electric	16	Off	15.0	177	1.7
8-May-15	Rock	2	Off	15.0	177	1.7
14-May-15	Nets	9	Off	11.5	161	1.7
14-May-15	Electric	11	Off	11.5	161	1.7
14-May-15	Rock	3	Off	11.5	161	1.7
15-May-15	Nets	8	On	10.9	163	1.7
15-May-15	Electric	11	On	10.9	163	1.7
15-May-15	Rock	4	On	10.9	163	1.7
21-May-15	Nets	7	On	12.8	183	1.4
21-May-15	Electric	6	On	12.8	183	1.4
21-May-15	Rock	3	On	12.8	183	1.4
22-May-15	Nets	7	Off	12.7	184	1.3
22-May-15	Electric	6	Off	12.7	184	1.3
22-May-15	Rock	3	Off	12.7	184	1.3

Supplementary Table 2. Number of PIT-tagged sea lamprey released in Chocolay River when the electric trap lead was off and on. Of the sea lamprey that moved upstream within 20 m of the electric lead (moved upstream), the percent that came within 1 m of the trap with electric lead (encountered electric trap), were captured in the trap with electric lead (captured in trap), moved downstream from the trap with electric lead (returned downstream), or escaped upstream of the trap with electric lead (escaped upstream of trap) are reported. PIT-tagged sea lamprey from different sources and released on different dates (**Supplementary Table 1**) are combined and reported together because variability in these responses were primarily explained by if the electric was off or on (not by source or other environmental factors).

Electric lead	Released	Moved upstream	Encountered electric trap	Captured in trap	Returned downstream	Escaped upstream of trap
Off	80	62%	4%	2%	2%	96%
On	78	59%	54%	33%	46%	22%

Supplementary Table 3. The source and number of PIT-tagged sea lamprey released in Bridgeland Creek according to date. Of the sea lamprey that moved upstream within 20 m of the electric lead (moved upstream), the percent that were captured in the trap with electric lead (captured in trap), moved downstream from the trap with electric lead (returned downstream), or escaped upstream of the trap with electric lead (escaped upstream of trap) are reported. During May 22nd, 2014, sea lamprey captured from the Echo River, Ontario, the trap with electric lead (VE-PDC Trap), and the trap upstream of the electric lead (Screen Trap) were released because of low sea lamprey availability from the Cheboygan River, MI. Recapture rates of sea lamprey obtained from these different sources did not vary substantially, although this was not tested statistically because of small sample sizes.

Release Date	Source	Released	Move upstream	Captured in trap	Return downstream	Escape upstream of trap
22-May-14	Screen Trap	25	95%	59%	0%	41%
22-May-14	VE-PDC Trap	25	87%	64%	0%	36%
22-May-14	Echo River	15	90%	44%	0%	56%
29-May-14	Cheboygan	100	96%	56%	0%	44%
5-Jun-14	Cheboygan	100	97%	77%	0%	23%
12-Jun-14	Cheboygan	99	93%	54%	1%	45%
16-Jun-14	Cheboygan	100	83%	42%	0%	58%
Combined	*All*	*464*	*91%*	*58%*	*0%*	*42%*
7-May-15	Cheboygan	100	93%	82%	0%	18%
12-May-15	Cheboygan	101	93%	77%	7%	16%
19-May-15	Cheboygan	100	96%	78%	8%	14%
27-May-15	Cheboygan	100	91%	76%	1%	23%
2-Jun-15	Cheboygan	100	97%	76%	5%	19%
9-Jun-15	Cheboygan	100	95%	63%	5%	32%
Combined	*All*	*601*	*94%*	*75%*	*4%*	*21%*

Supplementary Table 4. In Bridgeland Creek, Ontario, the number of individuals of each species captured in the trap with electric lead and the trap upstream of the electric lead during 2014 and 2015 and the percentage of non-targets that were dead when removed from the trap.

**2014**					
Species	Common Name	Electric Trap	% Dead	Upstream Trap	% Dead
Ambloplites rupestris	Rock Bass	337	40%	1129	2%
Ameiurus nebulosus	Brown Bullhead	92	58%	119	0%
Catostomus commersonii	White Sucker	13	77%	4	0%
Cottus bairdii	Mottled Sculpin	0	0%	0	0%
Esox lucius	Northern Pike	1	0%	2	50%
Etheostoma nigrum	Johnny Darter	1	0%	0	0%
Lepomis gibbosus	Pumpkinseed	3	33%	14	0%
Luxilus cornutus	Common Shiner	21	62%	63	3%
Oncorhynchus mykiss	Rainbow Trout	0	0%	0	0%
Orconectes sp.	Crayfish sp.	28	0%	4	0%
Perca flavescens	Yellow Perch	1	100%	0	0%
Rhinichthys cataractae	Longnose Dace	1	0%	2	0%
Salvelinus fontinalis	Brook Trout	2	50%	1	0%
Sander vitreus	Walleye	0	0%	1	0%
Semotilus atromaculatus	Creek Chub	11	73%	15	0%
	**Total**	**511**		**1354**	
**2015**					
Ambloplites rupestris	Rock Bass	1055	8%	491	1%
Ameiurus nebulosus	Brown Bullhead	43	0%	8	0%
Catostomus commersonii	White Sucker	11	55%	1	0%
Cottus bairdii	Mottled Sculpin	34	62%	5	25%
Esox lucius	Northern Pike	5	20%	3	0%
Etheostoma nigrum	Johnny Darter	3	33%	0	0%
Lepomis gibbosus	Pumpkinseed	4	25%	11	0%
Luxilus cornutus	Common Shiner	47	79%	2	0%
Oncorhynchus mykiss	Rainbow Trout	1	0%	0	0%
Orconectes sp.	Crayfish sp.	16	13%	14	14%
Perca flavescens	Yellow Perch	3	33%	1	0%
Rhinichthys cataractae	Longnose Dace	0	0%	2	2%
Salvelinus fontinalis	Brook Trout	5	0%	11	0%
Sander vitreus	Walleye	0	0%	0	0%
Semotilus atromaculatus	Creek Chub	35	23%	8	0%
	**Total**	**1262**		**557**	

Supplementary Table 5. In Bridgeland Creek, Ontario, species commonly captured each year in the trap upstream of the trap with electric lead since 1999. The column “1999-2013” provides the 95% confidence interval (alpha = 0.05) for how many of each species was captured in a given year. The columns “2014” and “2015” lists the number of individuals from each species captured during 2014 and 2015. Bolded numbers indicate those species whose catch during 2014 or 2015 fell below the 95% confidence interval for historic captures.

Species	Common Name	1999-2013	2014	2015
Ambloplites rupestris	Rockbass	491-1688	1129	491
Ameiurus nebulosus	Brown Bullhead	52-193	119	**8**
Catostomus commersonii	White Sucker	12-21	**4**	**1**
Lepomis gibbosus	Pumpkinseed	8-19	14	11
Luxilus cornutus	Common Shiner	20-95	63	**2**
Orconectes sp.	Crayfish sp.	9-45	**4**	14
Oncorhynchus mykiss	Rainbow Trout	2-8	**0**	**0**
Rhinichthys cataractae	Longnose Dace	2-18	2	2
Salvelinus fontinalis	Brook Trout	4-15	**1**	11
Semotilus atromaculatus	Creek Chub	31-86	**15**	**8**

Supplementary Table 6. Fish species in Bridgeland Creek, Ontario, that were PIT-tagged and released downstream the trap with the electric lead during 2015. Number tagged - number of each species tagged. Number detected - how many of the tagged fish were detected immediately downstream or upstream of the trap. Detected upstream - how many were detected upstream of the trap with the electric lead. Upstream during the day - how many of the fish detected upstream of the trap moved upstream during the day when the electric lead was off. Blocked at night – fish that were only detected downstream of the trap with electric lead during the night and therefore were presumed to have been blocked by the electric lead.

Species	Common Name	Number Tagged	Number Detected	Detected upstream	Upstream during day	Blocked at night
Ambloplites rupestris	Rock Bass	69	43	35	33	8
Ameiurus nebulosus	Brown Bullhead	9	3	1	1	2
Catostomus catostomus	Longnose Sucker	1	0	NA	NA	NA
Catostomus commersonii	White sucker	4	1	0	0	1
Esox lucius	Northern Pike	3	1	1	1	1
Oncorhynchus mykiss	Rainbow Trout	1	0	NA	NA	NA
